# BMC *Palliative Care* reviewer acknowledgement 2015

**DOI:** 10.1186/s12904-016-0090-y

**Published:** 2016-02-10

**Authors:** Dirk Krüger

**Affiliations:** BioMed Central, Floor 6, 236 Gray’s Inn Road, London, WC1X 8HB UK

## Abstract

The editors of BMC Palliative Care would like to thank all our reviewers who have contributed to the journal in Volume 14 (2015).

**Aziz Ansari**

USA

**Abraham Brody**

USA

**Carole Gardner**

UK

**Ai Oishi**

UK

**Albert J Kirshen**

Canada

**Anthony Mancini**

USA

**Angela Kydd**

UK

**Anna Thit Johnsen**

Denmark

**Anne Hofmeyer**

Australia

**Anne Wilkinson**

Australia

**Arif Kamal**

USA

**Sue Ronaldson**

Australia

**Jane Phillips**

Australia

**Ben White**

Australia

**Melissa Bloomer**

Australia

**Gregory Crawford**

Australia

**Geoffrey Mitchell**

Australia

**Maryann Street**

Australia

**Gaye Moore**

Australia

**Anna Chur-Hansen**

Australia

**Petra Graham**

Australia

**Tim Luckett**

Australia

**Martin Tattersall**

Australia

**Adam Walczak**

Australia

**Sarah Jeong**

Australia

**Barbara Head**

USA

**Baukje Miedema**

Canada

**Anja Declercq**

Belgium

**Joachim Cohen**

Belgium

**Benjamin Cheng**

Hong Kong

**Patria Berry**

USA

**Bert Leysen**

Belgium

**Juliano Ferreira Arcuri**

Brazil

**Carlos Paiva**

Brazil

**David Brent**

USA

**Susan McClement**

Canada

**Christopher Klinger**

Canada

**Kimberley Widger**

Canada

**Ebru Kaya**

Canada

**David Wright**

Canada

**Breffni Hannon**

Canada

**Lisa Barbera**

Canada

**Krista Noonan**

Canada

**Tamara Sussman**

Canada

**Lorraine Venturato**

Canada

**Christopher De Bono**

Canada

**Genevieve Thompson**

Canada

**Philippa Hawley**

Canada

**Robin Cohen**

Canada

**Vera Hirsh**

Canada

**David Streiner**

Canada

**Raymond Viola**

Canada

**Harold Siden**

Canada

**Kevin Weingarten**

Canada

**Michelle Howard**

Canada

**Carmen Houben**

Netherlands

**Catherine Lam**

USA

**Catriona Kennedy**

Ireland

**Cheryl Nekolaichuk**

Canada

**Christian Schulz**

Germany

**Christopher Klinger**

Canada

**Claudia Gamondi**

Switzerland

**Cathy Campbell**

USA

**Christopher Longo**

Canada

**Christine McPherson**

Canada

**Massimo Costantini**

Italy

**David N Korones**

USA

**Deborah Lewis**

UK

**Donna Wilson**

Canada

**Donnamaria Cortezzo**

USA

**Doralina Anghelescu**

USA

**Eleni Karasouli**

UK

**Eloise Carr**

Canada

**Elaine Morgan**

USA

**Felicity Hasson**

UK

**Fredrick Ashbury**

Canada

**Marilene Filbet**

France

**Freda Dekeyser Ganz**

Israel

**Bruno Gagnon**

Canada

**Jürgen in der Schmitten**

Germany

**Waldemar Siemens**

Germany

**Claudia Bausewein**

Germany

**Miriam van Buiren**

Germany

**Jan Gaertner**

Germany

**Steffen T Simon**

Germany

**Klaus Maria Perrar**

Germany

**Gloria Krahn**

USA

**Graeme Rocker**

Canada

**Geoff Wong**

USA

**Hiroki Fukahori**

Japan

**Andy Hau Yan Ho**

Hong Kong

**Hon Wai Benjamin Cheng**

Hong Kong

**Iwao Osaka**

Japan

**Anthony Staines**

Ireland

**Charles Normand**

Ireland

**Claire Donnellan**

Ireland

**Cinzia Brunelli**

Italy

**Paolo Gritti**

Italy

**Jan Schuling**

Netherlands

**Mitsunori Miyashita**

Japan

**Hiroya Kinoshita**

Japan

**Nobuhisa Nakajima**

Japan

**Takuya Shinjo**

Japan

**João Batista Santos Garcia**

Brazil

**Jayne Brown**

UK

**Garcia Jesusangel**

Spain

**Joe Low**

UK

**Karin Oechsle**

Germany

**Karen Detering**

Australia

**Katherine Peters**

USA

**Kathi Mooney**

USA

**Moens Katrien**

UK

**Kazuki Sato**

Japan

**Kirsten Wentlandt**

Canada

**Kathrin Milbury**

USA

**Lenzo Robijn**

Belgium

**Lorraine Holtslander**

Canada

**Laura Gilchrist**

USA

**Marike De Boer**

Netherlands

**Maho Aoyama**

Japan

**Maria Arantzamendi**

Spain

**Sandra Pereira**

Portugal

**Maxwell Vergo**

USA

**Marilyn Ballantyne**

Canada

**Melanie Smith**

USA

**Melissa Garrido**

USA

**Melissa Henry**

Canada

**Michael Teut**

Germany

**Michael McCrory**

USA

**Margaret O'Connor**

Australia

**Marc Sampedro Pilegaard**

Denmark

**Nicholas Hughes**

UK

**Natalie Evans**

Netherlands

**Martin Smalbrugge**

Netherlands

**Eleni Volakli**

Netherlands

**Annicka van**

Netherlands

**Anneke Leijntje Francke**

Netherlands

**Nick Hex**

UK

**Arnstein Finset**

Norway

**Peter Hudson**

Australia

**Anna Rahman**

USA

**Richard Harding**

UK

**Robin Ray**

Australia

**Susan Elizabeth**

UK

**Sallie-Anne Pearson**

Australia

**Sandra Schellinger**

USA

**Sarah Goodlin**

USA

**Saskia Juenger**

Germany

**Antonio Noguera**

Spain

**Srini Chary**

Canada

**Sumit Gupta**

USA

**Susie Lund**

UK

**Hans Thulesius**

Sweden

**Claudia Gambdi**

Switzerland

**Sriram Yennu**

USA

**Tessa Watts**

UK

**Hsiao-Ting Chang**

Taiwan

**Wen-Chi Chou**

Taiwan

**Tak Fung**

Canada

**Thomas Carroll**

USA

**Sema Sezgin Goksu**

Turkey

**Julia Downing**

Uganda

**Richard Powell**

Uganda

**Katherine Bristowe**

UK

**Ellen Henderson**

UK

**Libby Sallnow**

UK

**Helen Kerr**

UK

**Susan Ashton**

UK

**Jo Hockley**

UK

**Mark Howard**

UK

**Bob Whorton**

UK

**Victoria Allgar**

UK

**Padmanabhan Ramnarayan**

UK

**Caroline Barry**

UK

**Daniel Knights**

UK

**Victoria Simms**

UK

**Amy Gadoud**

UK

**Miguel Goncalves**

UK

**Paddy Stone**

UK

**Natalia Calanzani**

UK

**Sara Booth**

UK

**Mona Kanaan**

UK

**Olinda Santin**

UK

**Lorna Fraser**

UK

**Cara Bailey**

UK

**Charlotte Wilson**

UK

**Kristian Pollock**

UK

**Miranda Stacpoole**

UK

**Nathan Davies**

UK

**Chloe Chin**

UK

**Richella Ryan**

UK

**Wei Gao**

UK

**Jan Fish**

UK

**Sandra Varey**

UK

**Liz Bryan**

UK

**Sarah Yardley**

UK

**Neil McHugh**

UK

**Ruth Diver**

UK

**Irene Tuffrey-Wijne**

UK

**Nwamaka Eneanya**

USA

**Maxine De La Cruz**

USA

**Deborah Carr**

USA

**Sriram Yennu**

USA

**Joshua Jones**

USA

**Sherika Newman**

USA

**Eric Widera**

USA

**Timothy DeEulis**

USA

**Kelly Michelson**

USA

**Joshua Hauser**

USA

**Ladislav Volicer**

USA

**Max Vergo**

USA

**Linda Emanuel**

USA

**M. Jeanne Wirpsa**

USA

**Thomas Carroll**

USA

**Marcy Rosenbaum**

USA

**Susan Enguidanos**

USA

**Egidio Del Fabbro**

USA

**Thomas Leblanc**

USA

**Elaine Wittenberg**

USA

**Akhila Reddy**

USA

**Catherine Lam**

USA

**Elissa Miller**

USA

**Viv Lucas**

UK

